# Endothelial integrin α3β1 stabilizes carbohydrate-mediated tumor/endothelial cell adhesion and induces macromolecular signaling complex formation at the endothelial cell membrane

**DOI:** 10.18632/oncotarget.1837

**Published:** 2014-03-20

**Authors:** Olga V. Glinskii, Feng Li, Landon S. Wilson, Stephen Barnes, Kate Rittenhouse-Olson, Joseph J. Barchi, Kenneth J. Pienta, Vladislav V. Glinsky

**Affiliations:** ^1^ Research Service, Harry S. Truman Memorial Veterans Hospital, Columbia, MO; ^2^ Department of Pathology and Anatomical Sciences and the Department of Medical Pharmacology and Physiology, University of Missouri School of Medicine, Columbia, MO; ^3^ Department of Pharmacology and Toxicology and Targeted Metabolomics and Proteomics Laboratory, University of Alabama at Birmingham, Birmingham, AL; ^4^ Department of Biotechnical and Clinical Laboratory Sciences and the Department of Microbiology, University of Buffalo, Buffalo, NY; ^5^ Chemical Biology Laboratory, Center for Cancer Research, National Cancer Institute, Frederick National Laboratory for Cancer Research, Frederick, MD; ^6^ Department of Urology, The James Buchanan Brady Urological Institute, Departments of Oncology and Pharmacology and Molecular Sciences, The Johns Hopkins School of Medicine, Baltimore, MD

**Keywords:** tumor metastasis, adhesion, Thomsen-Friedenreich antigen, galectin, integrin

## Abstract

Blood borne metastatic tumor cell adhesion to endothelial cells constitutes a critical rate-limiting step in hematogenous cancer metastasis. Interactions between cancer associated carbohydrate Thomsen-Friedenreich antigen (TF-Ag) and endothelium-expressed galectin-3 (Gal-3) have been identified as the leading molecular mechanism initiating tumor/endothelial cell adhesion in several types of cancer. However, it is unknown how these rather weak and transient carbohydrate/lectin mediated interactions are stabilized. Here, using Western blot and LC tandem mass spectrometry analyses of pull-downs utilizing TF-Ag loaded gold nanoparticles, we identified Gal-3, endothelial integrin α3β1, Src kinase, as well as 5 additional molecules mapping onto focal adhesion pathway as parts of the macromolecular complexes formed at the endothelial cell membranes downstream of TF-Ag/Gal-3 interactions. In a modified parallel flow chamber assay, inhibiting α3β1 integrin greatly reduced the strength of tumor/endothelial cell interactions without affecting the initial cancer cell adhesion. Further, the macromolecular complex induced by TF-Ag/Gal-3/α3β1 interactions activates Src kinase, p38, and ERK1/2, pathways in endothelial cells in a time- and α3β1-dependent manner. We conclude that, following the initial metastatic cell attachment to endothelial cells mediated by TF-Ag/Gal-3 interactions, endothelial integrin α3β1 stabilizes tumor/endothelial cell adhesion and induces the formation of macromolecular signaling complex activating several major signaling pathways in endothelial cells.

## INTRODUCTION

Metastasis is a major cause of cancer-related morbidity and mortality. The initial arrest of blood borne metastatic tumor cells in distant organ microvasculature constitutes an important rate-limiting step in hematogenous cancer metastasis [1-3]. Consequently, there has been a substantial surge in recent years in research aiming to identify the molecules mediating tumor cell adhesion to endothelial cells [4-13]. Among others, interactions between cancer-associated carbohydrate Thomsen-Friedenreich antigen (TF-Ag), core 1 disaccharide Galβ1-3GalNAc expressed on 80-90% of all adenocarcinomas [14-16], and endothelium expressed ~30kDa β-galactoside binding lectin galectin-3 (Gal-3) have been identified as the leading molecular mechanism mediating the initial stages of adhesion between metastatic cells and endothelium in multiple types of cancer including breast [9, 17-19], prostate [9, 17, 20], colon [7] and pancreas [6]. The multifaceted role for Gal-3 in tumor metastasis was first established by the pioneering works from the group of Dr. Avraham Raz [21-24]. Subsequently, it has been demonstrated that, while cancer cell expressed Gal-3 is involved in regulation of tumor cell apoptosis and homotypic aggregation [9, 21, 24, 25] and may even serve as a complementary serum marker in prostate cancer [26], it is endothelium expressed Gal-3 that mediates metastatic cell adhesion to the endothelium via interactions with cancer associated TF-Ag [9, 17-20]. Specifically, it has been shown that in the process of metastasis-associated endothelial activation TF-Ag expressed on either circulating tumor-associated glycoproteins or blood borne metastatic cells induces Gal-3 translocation to and clustering at the endothelial cell membrane [17, 27]. Subsequently, TF-Ag expressing cancer cells interact with cell surface clustered endothelial Gal-3 to initiate metastatic cell arrest in distant organ vasculature [9, 17-20]. However, these carbohydrate/lectin mediated adhesive interactions are rather weak and transient in nature. Without further stabilization transiently adhered cancer cells could be dislodged by the shear force of the flowing blood resulting in the interruption of the metastatic cascade. Indeed, Gal-3 does not contain a transmembrane domain and, therefore, lacks means of being anchored at the endothelial cell surface without the involvement of additional molecules capable of providing such anchorage. However, it is currently unknown which molecules stabilize tumor/endothelial cell adhesion downstream of TF-Ag/Gal-3 interactions.

Recently, it has been shown that, similarly to cancer-associated TF-Ag, NG2 proteoglycan produced by pericytes mediates Gal-3 translocation to and clustering at the endothelial cell membrane followed by the mobilization of endothelial α3β1 integrin, which physically interacts with Gal-3 scaffolds [28]. This information led us to hypothesize that endothelial integrins such as α3β1 could be likewise mobilized to Gal-3 clustered at the endothelial cell membrane during TF-Ag mediated metastatic cell adhesion to the endothelium and stabilize initial transient carbohydrate/lectin mediated interactions. In the present study, we report that endothelial integrin α3β1 does indeed interact directly with TF-Ag/Gal-3 complexes and significantly increases the strength of tumor/endothelial cell adhesion. Further, upon tumor cell/endothelial cell interactions, we detected activation of several major signaling pathways downstream of endothelial α3β1 suggesting that TF-Ag/Gal-3 mediated interactions induce complex signaling crosstalk in endothelial cells, which could be potentially targeted for therapeutic purposes.

## RESULTS

### Endothelial integrin α3β1 associates physically with TF-Ag/Gal-3 clusters and induces the formation of focal adhesion type macromolecular signaling complexes

**Figure1:**
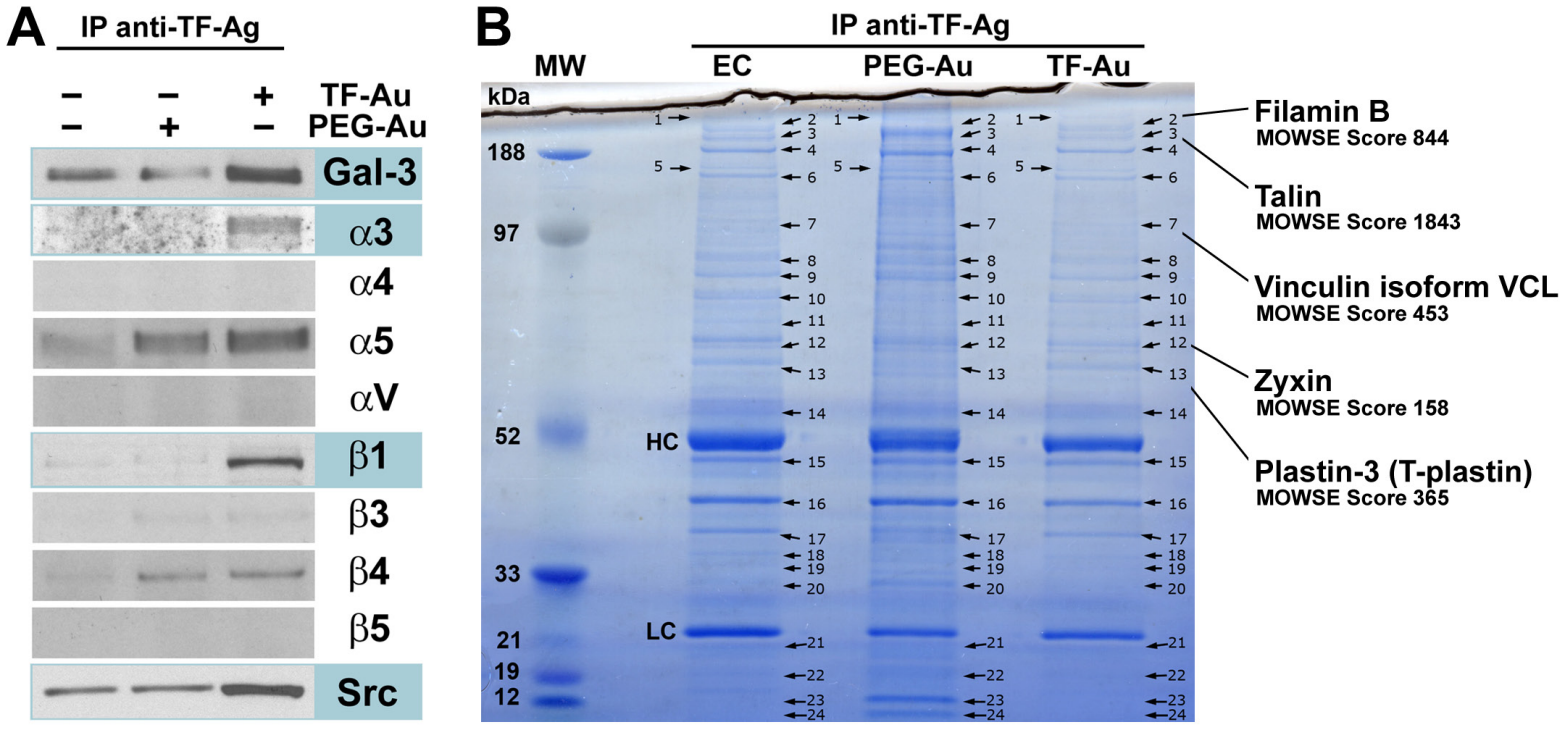
Macromolecular complexes formed at the endothelial cell membrane downstream of TF-Ag/Gal-3 interactions A, Western blot analysis of the integrin molecules and other proteins interacting with TF-Au nanoparticles at the endothelial cell surfaces. Note specific interactions of Gal-3 and integrin subunits α3, and β1 with TF-Au nanoparticles indicative of α3β1 interactions with TF-Ag/Gal-3 complexes. The Src kinase, a principal signal transduction molecule downstream of α3β1 integrin also interacts specifically with TF-Au nanoparticles. B, Using LC-tandem mass spectrometry, additional 5 proteins mapping onto focal adhesion pathway [filamin B, β (Mr 263,856); talin (Mr 269,486); vinculin isoform VCL (Mr 116,649); zyxin (Mr 61238); and plastin-3/T-plastin (Mr 70,391] were identified in bands 2, 3, 7, 12, and 13 respectively in TF-Au, but not PEG-Au or control endothelial cell (EC) pull-downs. In B, bands corresponding to heavy chain and light chain of the anti-TF-Ag antibody used for a pull-down denoted as HC and LC respectively. The experiments were performed three times for Western analysis and twice for LC-tandem mass spectrometry with the same results.

Previously, we demonstrated that in the process of metastasis-associated endothelium activation TF-Ag expressed on circulating tumor-associated glycoproteins or blood borne metastatic cells induces Gal-3 translocation to and clustering at the endothelial cell outer membranes [17, 27]. Subsequently, TF-Ag expressing tumor cells interact with cell surface clustered endothelial Gal-3 to initiate metastatic cell adhesion to the vascular wall [9, 17-20, 27]. To identify integrin molecules and other proteins interacting with TF-Ag/Gal-3 complexes at the endothelial cell membrane we have performed a pull-down assay using gold nanoparticles bearing multiple TF-Ag moieties (TF-Au) covalently attached to the particles through the polyethylene glycol (PEG) linker and control nanoparticles, on which PEG linkers are terminated with the OH groups (PEG-Au) as described in Materials and Methods. Consistent with TF-Ag interactions with Gal-3 scaffolds, Western blot analysis of the pull-down isolates confirmed the presence of Gal-3 in TF-Au samples (Fig. 1, A). Probing the membranes with the panel of antibodies directed against α3, α4, α5, αV, β1, β3, β4, and β5 integrins revealed the presence of α3 and β1 integrin subunits (i.e. α3β1 integrin) in TF-Au, but not in untreated endothelial cell or PEG-Au samples (Fig. 1, A) indicative of α3β1 interaction with TF-Ag/Gal-3 complexes. The signals for α5 and β4 integrins were clearly identifiable as well (Fig. 1, A). However, they were present in both TF-Au and PEG-Au samples suggesting that they were pulled down due to nonspecific interactions with PEG. In addition, a principal signal transducing molecule downstream of α3β1 integrin, the Src kinase, was also present in TF-Au pull-downs (Fig. 1, A). Next, individual bands were cut from NuPAGE 4-12% gradient Bis-Tris gels stained with modified colloidal Coomassie Blue G-250 (Fig. 1, B) and analyzed by LC-tandem mass spectrometry following in gel digestion and tryptic peptide extraction. Using this approach, we identified with high fidelity (MOWSE scores ranging from 158 to 1843) additional 5 proteins, which were present in TF-Au samples only: filamin B, β (Mr 263,856); talin (Mr 269,486); vinculin isoform VCL (Mr 116,649); zyxin (Mr 61238); and plastin-3/T-plastin (Mr 70,391) in bands 2, 3, 7, 12, and 13 respectively (Fig. 1, B). Remarkably, all of these 5 proteins map onto focal adhesion pathway. Taken together, these results strongly suggest that interactions of tumor-associated TF-Ag with endothelial Gal-3 mobilize endothelial α3β1 integrin, which associates physically with Gal-3 scaffolds and induces the formation of focal adhesion type macromolecular signaling complexes at the endothelial cell membrane. Thus, our next two questions were: (i) Whether α3β1 integrin engagement stabilizes tumor/endothelial cell adhesion; and (ii) Whether these interactions induce major signal transduction pathways in endothelial cells downstream of α3β1 integrin?

### Integrin α3β1 stabilizes tumor/endothelial cell adhesion

**Figure 2:**
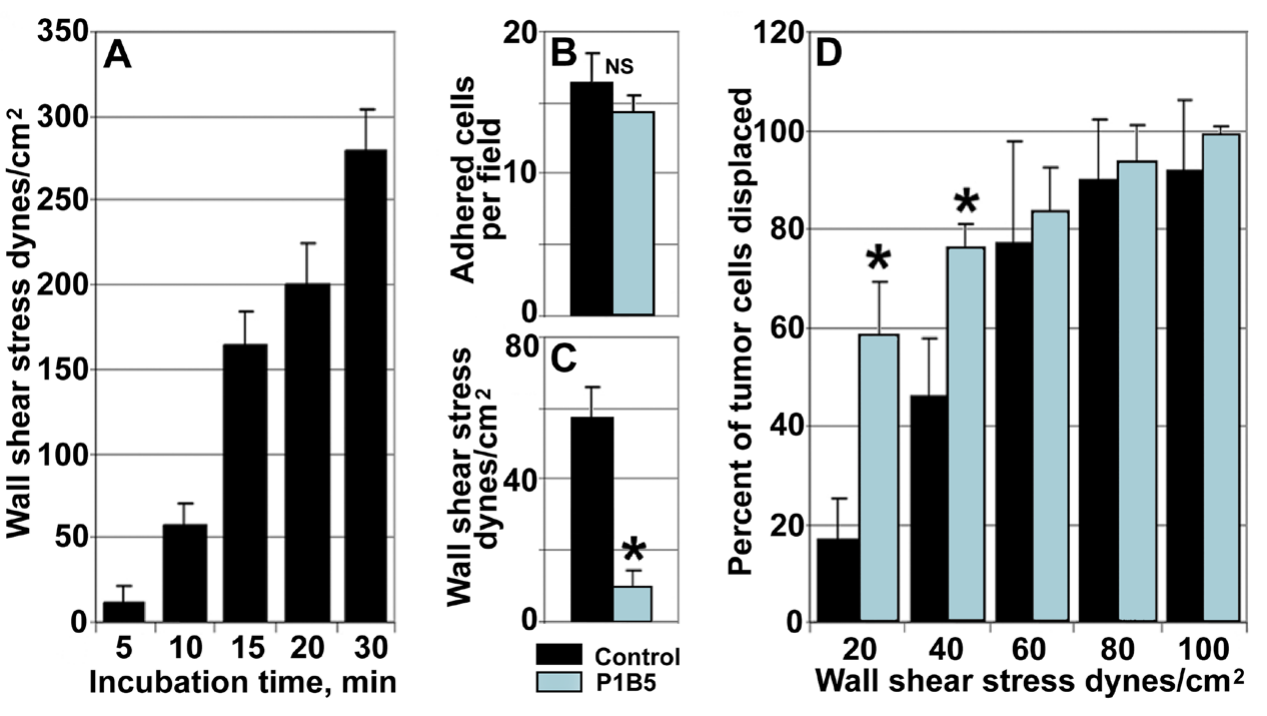
Endothelial integrin α3β1 stabilizes tumor/endothelial cell adhesion A, Tumor/endothelial cell adhesion undergoes rapid stabilization. Note rapid increase in a wall shear stress required to displace 50% of stably adhered tumor cell after 10, 15, 20, and 30 min of incubation with endothelial monolayers. B, Function blocking anti-α3β1 antibody P1B5 (blue bar) does not affect the initial adhesion of tumor cells to the endothelium after 10 min of incubation compared with control IgG (black bar). C and D, Anti-α3β1 function blocking antibody P1B5 inhibits tumor/endothelial cell adhesion stabilization. C, Compared with control IgG (black bar), anti-α3β1 antibody P1B5 (blue bar) reduces significantly the wall shear stress required to displace 50% of stably adhered tumor cell after 10 min of incubation. D, Compared with control IgG (black bars), anti-α3β1 antibody P1B5 (blue bars) increases significantly the percent of tumor cells displaced by the wall shear stress of 20 and 40 dynes/cm2 after 10 min of incubation. In A thorough D, data presented as means ± STDEV; *denotes statistical significance (p<0.05)

Here, we have employed a modified parallel flow chamber assay to analyze temporal dynamics of changes in the strength of adhesion between metastatic tumor cells and endothelial monolayers. The assay has been designed to allow tumor cells to interact with endothelial monolayers for various time periods under static conditions followed by the application of the increasing wall shear force of a defined magnitude in a parallel flow chamber to displace stably adhered tumor cells. In control experiments (Fig. 2, A), we observed a significant time-dependent increase in a wall shear force necessary to displace 50% of stably adhered tumor cells rapidly reaching the values far exceeding physiological wall shear stress (4-8 dynes/cm^2^) at which tumor cell adhesion to the vascular wall occurs. In our experiments, after 10 min of incubation the wall shear stress displacing 50% of tumor cells was 56.4 ± 12.0 dynes/cm^2^ and it was reaching upwards of 250 dynes/cm^2^ after 30 min (Fig. 2, A). These results indicate that tumor/endothelial cell adhesion undergoes rapid stabilization resulting in a significant increase in the adhesion forces between the two cell types. To investigate whether α3β1 integrin is responsible for the observed tumor/endothelial cells adhesion stabilization, we used anti-α3β1 function blocking antibody P1B5 [29]. Performing experiments in the presence of 10 μg/ml of anti-α3β1 did not change significantly the number of stably adhered tumor cells compared with control mouse IgG (Fig. 2, B). However, the wall shear stress required to displace 50% of tumor cells has been significantly (p<0.05) reduced by anti-α3β1 from 56.4 ± 12.0 dynes/cm^2^ to 9.2 ± 4.3 dynes/cm^2^ (Fig. 2, C). Further, the percent of tumor cells displaced after 10 min of incubation by the flow inflicting wall shear stress of 20 and 40 dynes/cm^2^ has been also increased significantly by anti-α3β1 antibody (Fig. 2, D). Taken together, these results demonstrate that α3β1 does not affect the initial (carbohydrate-mediated) tumor cell adhesion to endothelial cells, but plays a critical role in stabilizing tumor/endothelial cell adhesive interactions.

### Tumor/endothelial cell interactions activate major signaling pathways in endothelial cells in α3β1 dependent manner

**Figure 3:**
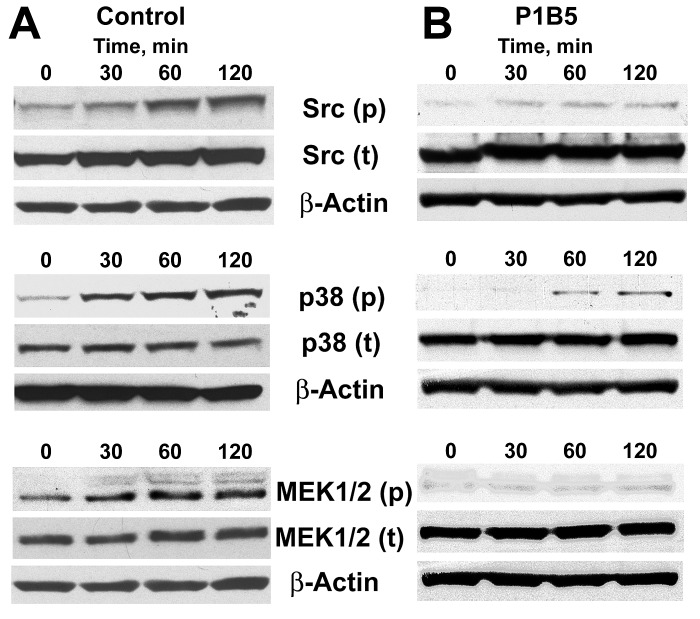
Activation of major signaling pathways in endothelial cells downstream of integrin α3β1 A, Time-dependent activation of Src, p38, and MEK1/2 in endothelial cells following their interaction with tumor cells for the indicated time periods. B, Anti-α3β1 function blocking antibody P1B5 inhibits time-dependent activation of Src, p38, and MEK1/2 in endothelial cells following their interaction with tumor cells for the indicated time periods. The experiments were repeated at least three times for each setting with similar results.

As our TF-Gold pull-down experiments revealed, in addition to Gal-3 and α3β1 integrin, the presence of the Src kinase (a principal signal transduction molecule acting downstream of α3β1), as well as of 7 other proteins mapping onto focal adhesion pathway (Fig. 1) in TF-Au samples, our next question was whether tumor/endothelial cell adhesive interactions activate major signaling pathways in endothelial cells downstream of α3β1 integrin. Typically, α3β1 induces via Src kinase major MAPK (p38 and ERK1/2) signal transduction pathways. Indeed, Western blot analysis of the endothelial cell lysates following tumor/endothelial cell co-culture experiments demonstrated time-dependent activation of Src, p38 MAPK, and MEK1/2 in endothelial cells, when they interact with tumor cells (Fig. 3, A). Performing the same experiments in the presence of the function-blocking anti-α3β1 antibody P1B5 abolished time-dependent activation of these signaling pathways (Fig. 3, B). These results indicate that tumor/endothelial cell interactions induce activation of Src and major MAPK signal transduction pathways (p38 and ERK1/2) in endothelial cells in a time-dependent manner downstream of endothelial α3β1 integrin.

## DISCUSSION

The results presented in this study add important new insights to our understanding of the extremely complex multi-step process of hematogenous cancer metastasis. They demonstrate that, following the initial transient adhesive interactions between metastatic tumor cells and vascular endothelium mediated by cancer-associated TF-Ag and endothelium expressed Gal-3, endothelial integrin α3β1 physically associates with TF-Ag/Gal-3 complexes at the endothelial cell surfaces and stabilizes tumor/endothelial cell adhesion. This stabilization results in a rapid increase of the strength of adhesion between endothelial and tumor cells reaching the values far exceeding physiological shear forces acting upon adhered metastatic cells in the circulation. Without such stabilization, metastatic tumor cells attached to the vascular wall via weak carbohydrate/lectin interactions will be dislodged by the flowing blood and metastatic cascade will be interrupted. This makes the endothelial α3β1 integrin an attractive therapeutic target for controlling metastatic spread of cancer.

Further, in addition to stabilizing tumor/endothelial cell adhesive interactions, endothelial α3β1 engagement triggers the formation of the focal adhesion type macromolecular signaling complexes at the endothelial cell membrane and time-dependent phosphorylation of Src, p38, and MEK1/2 in endothelial cells downstream of α3β1 indicative of the activation of p38 and ERK1/2 signal transduction pathways. As these pathways regulating important cellular functions related to cell survival, motility, and proliferation share multiple downstream targets, these results show that tumor/endothelial cell interactions induce complex crosstalk between major MAPK signal transduction pathways, which could have a profound effect on endothelial cell behavior. The biological outcomes of this crosstalk in a context of cancer metastasis and its potential effects on subsequent steps of the metastatic cascade such as tumor cell transendothelial migration and extravasation are currently under investigation and could yield yet additional therapeutic targets for anti-metastatic interventions. For example, previous studies from the group of Dr. M. Sharon Stack have shown that, when both p38 and ERK1/2 MAPK signaling pathways are simultaneously activated in tumor cells downstream of α3β1 integrin, the resulting outcome of the interplay between the two pathways is the activation of tumor cell MMP system in a p38 dependent manner [30]. Whether the same scenario takes place when p38 and ERK1/2 pathways are activated downstream of α3β1 in endothelial cells as a consequence of tumor/endothelial cell adhesion, and whether endothelial p38, ERK1/2, and MMPs could serve as molecular targets for anti-metastatic therapies could be of paramount interest.

Previously, we demonstrated that TF-Ag mimicking and TF-Ag blocking inhibitors significantly reduce breast and prostate carcinoma metastasis *in vivo* by impeding the initial TF-Ag/Gal-3 mediated tumor cell adhesion to the endothelium [9, 19, 20]. Recently, yet another group used natural TF-Ag expressing glycopeptide TFD100 isolated from Atlantic cod to inhibit TF-Ag/Gal-3 mediated tumor/endothelial cell adhesion and ultimately PC-3 metastasis in vivo [31]. Inhibiting simultaneously additional subsequent steps of tumor metastasis mediated by endothelial integrins will increase dramatically our ability to control hematogenous spread of cancer. With these regards, it appears that endothelial α3β1, Src, and MAP kinases could serve as valuable therapeutic targets. Further, the means for therapeutic targeting of the same signaling pathways in tumor cells are actively developed [32]. Identifying relevant molecular targets for anti-metastatic therapies located not in tumor cells, but in the target organ vasculature may present a new paradigm for controlling and preventing cancer metastasis.

## MATERIALS AND METHODS

### Cell lines and antibodies

Metastatic human prostate carcinoma PC-3 cells (ATCC, Rockville, MD) were maintained as monolayer cultures using RPMI-1640 media supplemented with 10% FBS in a 5% CO2 humidified incubator. Human umbilical vein endothelial cells, HUVEC, (Life Technologies, Grand Island, NY) were cultured using Basal Medium 200 (Life Technologies) supplemented with low serum growth supplement containing FBS (2% v/v final concentration), hydrocortisone, human fibroblast growth factor, heparin, and human epidermal growth factor. The following antibodies were used in this study: anti-TF-Ag produced by JAA-F11 hybridoma [33]; anti-Gal-3 produced by TIB-166 hybridoma (ATCC); anti-integrin α3 (clone P1B5, EMD Millipore, Billerica, MA); Integrin Antibody Sampler Kit (#4749) including antibodies against integrins α4, α5, αV, β1, β3, β4, and β5; anti-phospho-Src (#2101); anti-Src (#2123); anti-phospho-p38 (#9211); anti-p38 (#9212); anti-phospho-MEK1/2 (#9121); anti-MEK1/2 (#9122); anti-phospho-Akt (#9271); anti-Akt (#9272) all from Cell Signaling, Danvers, MA; anti-β-Actin (ab8227) from Abcam, Cambridge, MA.

### TF-Gold pull-down

Gold nanoparticles carrying multiple TF antigen epitopes (TF-Au) covalently attached to the particles through the polyethylene glycol (PEG) linker and control nanoparticles, on which PEG linkers are terminated with the OH groups (PEG-Au) and exhibiting excellent solubility and stability were prepared as previously described [34]. Confluent endothelial cell monolayers grown for 6 days in collagen-coated T-150 flasks were treated for 60 min with TF-Au or PEG-Au nanoparticles (250 μl of 4 mg/ml solution diluted in 20 ml of complete RPMI-1640 media containing 5% FBS), while endothelial cells treated with RPMI-1640 media containing 5% FBS only served as an additional control. Next, unbound nanoparticles were washed away by rinsing cultures twice with ice cold PBS; the cells were lysed using CelLytic-M mammalian cell lysis/extraction reagent (Sigma, Saint Louis, MO) supplemented with protease inhibitor cocktail (Sigma) and subjected to immunoprecipitation (IP) using JAA-F11 anti-TF-Ag antibody and protein A agarose. The immunoprecipitates were resolved on NuPAGE 4-12% gradient Bis-Tris gels (Invitrogen, Carlsbad, CA) and used either for Western blot analysis or LC-tandem mass spectrometry to identify precipitated proteins.

### Western blot analysis

For TF-Gold pull-down analysis, TF-Au, PEG-Au, and untreated endothelial cell pull-down isolates were resolved on NuPAGE 4-12% gradient Bis-Tris gels (Invitrogen), and transferred to a nitrocellulose membrane (Invitrogen). The membranes were probed with primary antibodies against Gal-3, α3, α4, α5, αV, β1, β3, β4, and β5 integrins, and Src kinase (see antibody list above) in conjunction with corresponding HRP-conjugated secondary antibodies and enhanced chemiluminescent (ECL) detection.

Endothelial cells from co-culture experiments (see below) were lysed using CelLytic M buffer with protein inhibitor cocktail (Sigma). Protein concentrations were determined using Protein Assay kit (Bio-Rad). Equal amounts of the protein from each sample (30 μg) were resolved on a NuPAGE 4-12% gradient Bis-Tris gels (Invitrogen), and transferred to a nitrocellulose membranes (Invitrogen). The membranes were sequentially probed with primary antibodies directed against phosphorylated and total Src, p38, MEK1/2, and Akt (see antibody list above) in conjunction with corresponding HRP-conjugated secondary antibodies and enhanced chemiluminescent (ECL) detection. Anti-β-Actin antibody (Abcam, Cambridge, CA) was used to control loading. The experiments were performed at least three times for each setting with same results.

### Mass spectrometry analysis

TF-Au, PEG-Au, and untreated endothelial cell pull-down isolates resolved on NuPAGE 4-12% gradient Bis-Tris gels were stained with modified colloidal Coomassie Blue G-250 [35] and 25 individual bands were cut by hand from each lane (Fig. 1, B). In gel digestion using mass spectrometry grade trypsin gold (Promega, Madison, WI), peptide extraction, and LC-tandem mass spectrometry analyses on the Applied Biosystems-MDS-Sciex (Concorde, Ontario, Canada) 4000 Qtrap mass spectrometer were performed as previously described [36]. The tandem mass spectrometry data were processed for protein identifications using an in-house MASCOT search engine version 4.2 (Matrix Science, Boston, MA) using the Human NCBInr protein database and one missed protease cleavage site. Variable modifications were allowed for oxidized methionines and a fixed modification for carbamidomethylated cysteines. Significant proteins hits were any protein(s) that had at least one individual peptide sequence score of >40. The experiments were repeated twice with same results.

### Modified parallel flow chamber experiments

A modification of the parallel flow chamber assay was developed to enable the analysis of changes in strength of adhesion between tumor and endothelial cells. Briefly, endothelial cells were grown until confluent in 35 mm tissue culture dishes. Next, 1 ml of a single tumor cell suspension (5 x 10^5^ cell/ml) prepared immediately prior the experiment using nonenzymatic cell dissociation reagent (Sigma) was applied on top of the endothelial monolayer and allowed to interact with endothelial cells for various (5, 10, 15, 20, 30 min) time periods at 37°C in a CO_2_ incubator (static adhesion phase). After that, unbound tumor cells were gently washed away, a parallel flow chamber deck (Glycotech, Rockville, MD) was mounted on top of the 35 mm dish on a stage of the inverted video microscope, and gradually increasing flow of a displacement media was initiated using a precision syringe pump KDS210 (KD Scientific, New Hope, PA) generating a wall shear force of a defined magnitude (parallel flow phase), while the process of cancer cell detachment and displacement was monitored and video recorded. Based on these experiments, temporal dynamics of tumor-endothelial cell adhesion stabilization were determined including: 1). The relationship between the time of tumor cell interaction with endothelium and a wall shear force necessary to displace 50% of tumor cells, and 2). The relationship between the wall shear force applied and a percentage of adherent tumor cells displaced. The experiments were performed at least three times for each setting and the data presented as means ± STDEV.

### Tumor cell/endothelial cell co-cultures

To investigate the changes in phosphorylation and expression of the proteins of interest in endothelial cells when they interact with tumor cells, an experimental system was developed enabling a fast and efficient separation of endothelial and cancer cells following their interaction. To achieve this, we took advantage of the observation that endothelial cells, after growing on a collagen support for 6-7 days, become strongly attached to the collagen coated plastic and fairly resistant to the action of cell dissociation reagents, whereas tumor cells interacting with endothelium for a relatively short time (0-120 min) could be easily dislodged using non-enzymatic cell dissociation solution (Sigma). Thus, we performed series of experiments, in which 5 x 10^5^ PC-3 cells stably transfected with GFP (for controlling the efficiency of tumor cell separation from endothelial cells) interacted for various periods of time (0, 30, 60, 120 min) with endothelial monolayers growing for 7 days in collagen-coated T150 flasks. After that tumor cells where quickly separated from endothelial monolayers using nonenzymatic cell dissociation reagent (Sigma), harvested, and frozen at -70°C for future analysis, while endothelial cells were lysed on plastic using cell lysis buffer with protein inhibitor cocktail (Sigma) and analyzed by Western blot as described above. The experiments were performed at least three times for each setting.
